# Identification of genes regulating migration and invasion using a new model of metastatic prostate cancer

**DOI:** 10.1186/1471-2407-14-387

**Published:** 2014-05-30

**Authors:** Jacqueline Banyard, Ivy Chung, Matthew Migliozzi, Derek T Phan, Arianne M Wilson, Bruce R Zetter, Diane R Bielenberg

**Affiliations:** 1 Vascular Biology Program, Boston Children’s Hospital, Karp Family Research Laboratories, 300 Longwood Avenue, 02115 Boston, MA, USA; 2 Department of Surgery, Harvard Medical School, 02115 Boston, MA, USA; 3 Current address; Department of Pharmacology, Faculty of Medicine, UM Cancer Research Institute, University of Malaya, 50603 Kuala Lumpur, Malaysia

**Keywords:** Prostate cancer, Invasion, Migration, Metastasis, Angiogenesis, Lymphangiogenesis, Lymph node, EpCAM, Integrin, Beta4, uPA, New model

## Abstract

**Background:**

Understanding the complex, multistep process of metastasis remains a major challenge in cancer research. Metastasis models can reveal insights in tumor development and progression and provide tools to test new intervention strategies.

**Methods:**

To develop a new cancer metastasis model, we used DU145 human prostate cancer cells and performed repeated rounds of orthotopic prostate injection and selection of subsequent lymph node metastases. Tumor growth, metastasis, cell migration and invasion were analyzed. Microarray analysis was used to identify cell migration- and cancer-related genes correlating with metastasis. Selected genes were silenced using siRNA, and their roles in cell migration and invasion were determined in transwell migration and Matrigel invasion assays.

**Results:**

Our *in vivo* cycling strategy created cell lines with dramatically increased tumorigenesis and increased ability to colonize lymph nodes (DU145LN1-LN4). Prostate tumor xenografts displayed increased vascularization, enlarged podoplanin-positive lymphatic vessels and invasive margins. Microarray analysis revealed gene expression profiles that correlated with metastatic potential. Using gene network analysis we selected 3 significantly upregulated cell movement and cancer related genes for further analysis: EPCAM (epithelial cell adhesion molecule), ITGB4 (integrin β4) and PLAU (urokinase-type plasminogen activator (uPA)). These genes all showed increased protein expression in the more metastatic DU145-LN4 cells compared to the parental DU145. SiRNA knockdown of EpCAM, integrin-β4 or uPA all significantly reduced cell migration in DU145-LN4 cells. In contrast, only uPA siRNA inhibited cell invasion into Matrigel. This role of uPA in cell invasion was confirmed using the uPA inhibitors, amiloride and UK122.

**Conclusions:**

Our approach has identified genes required for the migration and invasion of metastatic tumor cells, and we propose that our new *in vivo* model system will be a powerful tool to interrogate the metastatic cascade in prostate cancer.

## Background

Prostate cancer affects 1 in 6 males in their lifetime, and is the second leading cause of cancer death in men in the U.S. [1]. Almost 2.8 million men are currently living with a diagnosis of prostate cancer [2], yet the ability to discern whose cancer will progress to metastatic disease remains a challenge. A better understanding of the metastatic process could lead to enhanced prognostic ability and subsequent improvements in patient care and outcome. Cancer cells can escape the primary tumor via blood vessels or lymphatic vessels and travel to distant organs. The presence of tumor cell-positive lymph nodes from biopsy indicates the tumor has already spread from the primary site. Lymph node metastasis is an important prognostic indicator in many cancers, such as breast, melanoma and prostate [3-6]. Lymph node metastasis correlates with poor prognosis in prostate cancer, as compared to those without lymph node involvement [7]. Even before evidence of lymph node metastasis, lymphovascular invasion (LVI), defined as the unequivocal presence of tumor cells within an endothelium-lined space, can act as an independent risk factor in prostate cancer [5]. Since all lymphatic drainage eventually empties into the venous system, tumor extravasation into lymphatic vessels may lead to more widespread metastasis via the vascular circulatory system to distant organs like bone [8,9].

As many patients now opt for an active surveillance or ‘watchful waiting’ period during the management of organ-confined disease [10,11], the development of new biomarkers and therapeutic options is greatly needed. The identification of genes important in the metastatic cascade may facilitate our development of such therapies.

Animal models of metastasis are important tools that allow us to interrogate steps in this process. Spontaneous and experimental models of metastasis in mice have allowed us to discover and analyze new genes and biomarkers and to test anti-cancer drugs within complex microenvironments. Studies have shown that when human cancer cell xenografts are implanted into the orthotopic site, as compared to an ectopic (usually subcutaneous) site, enhanced tumorigenicity and metastasis followed [12-14]. The microenvironment is well documented to influence tumor cell behavior and is capable of stimulating or repressing cell plasticity, proliferation, migration and invasion [15-17]. Orthotopically implanted tumor cells and their spontaneously metastasizing counterparts are exposed to many of the same environmental influences and selective pressures that human prostate cancer cells undergo in the prostate and lymph nodes. In addition, human xenografts allow one to interrogate the efficacy of human-specific drugs such as proteins (eg, interferons) or antibodies (eg, bevacizumab). Xenograft models provide a complement to genetically engineered mouse models which develop over a longer time and reside in an immunocompetent host but do not always capture all aspects of human cancer.

*In vivo* cycling of cancer cells has been demonstrated to be a useful method to select for highly aggressive cell lines. The human prostate cancer cell lines, PC-3 and LNCaP, were previously cycled *in vivo* to select for highly metastatic variants from sentinel lymph node metastasis [12,18]. These human cancer models have proven highly beneficial to the prostate cancer research community [19]. Herein, we describe a similar method to create a novel prostate cancer model developed in our laboratory using the DU145 human prostate cancer cell line. Originally isolated by Stone, et. al., from a human brain metastasis, DU145 is a “classical” and widely-used prostate cancer cell line [20]. DU145 cells do not express detectable levels of prostate specific antigen and are not hormone sensitive.

This report describes the development and characterization of this model and our studies investigating molecular changes that correlate with metastatic potential.

## Methods

### Cell culture and transfection

DU145 human prostate cancer cells were obtained from ATCC (HTB-81) and maintained in high glucose DMEM with 10% fetal bovine serum (FBS), 1% glutamine, penicillin and streptomycin (GPS), and 1% sodium pyruvate (Invitrogen, Carlsbad, CA). Phase contrast microscopy was performed using a TE2000 microscope (Nikon) and RT SPOT camera with SPOT Advanced v4.0.9. software (Diagnostic Instruments, Inc., Sterling Heights, MI). Cells were transfected with siRNA using SilentFect (Biorad) in Opti-MEM I Reduced Serum Medium (Invitrogen), incubated for 4 hours, media changed, and cells used for assays at 48-72 hr. siRNAs were obtained from Thermo Scientific: ON-TARGETplus non-targeting control siRNA pool (D-001818-10-05), ON-TARGETplus human EPCAM siRNA pool (L-004568-01-0005), ON-TARGETplus human PLAU siRNA (L-006000-00-0005), ON-TARGETplus human ITGB4 siRNA pool (L-008011-00-0005). EPCAM and ITGB4 siRNAs were used at 30nM and PLAU siRNA used at 90nM for effective knockdown without toxicity.

### Cell migration, invasion and proliferation assays

Cell migration was measured using Corning transwell inserts (BD Biosciences) with 8.0 μm pore polycarbonate membrane. Membranes were coated with Collagen I (BD Biosciences) at 100 μg/ml. 1% FBS in DMEM was used in the lower wells as chemoattractant. Cells were trypsinized, trypsin inactivated with soybean trypsin inhibitor and washed in DMEM. 6×10^4^ cells were added to the top transwell chamber and allowed to migrate for 4 hours. Cells were fixed and stained with Diff-Quik (Fisher Scientific) and a cotton swab used to remove non-migrated cells from the upper chamber. Migrated cells were counted in 3–5 fields/well with 2–3 wells/condition. Cells were used for experiments 48 hours after transfection. For invasion assays, BD BioCoat Matrigel Invasion Chambers, with 8.0 μm pore PET membrane in 24-well cell culture inserts (BD Biosciences) were used with 5% FBS as the chemoattractant. Cells were allowed to invade for 12 hours and were fixed, stained and counted as described above. For uPA inhibitor experiments, cells were treated with 0.1% DMSO vehicle, 10 μM amiloride or UK122 (EMD Millipore, Billerica, MA). *In vitro* cell number was measured using CyQUANT Cell Proliferation Assay kit (Life Technologies). Cells were plated in a 96 well plate at 2.5×10^3^ cells per well and incubated for 1–4 days. Plates were frozen and processed together at the end of the experiment. Fluorescent signal correlated with cell number and was measured with 450 nm excitation and 520 nm emission filters.

### Western blot analysis

Whole cell lysates were collected in modified RIPA buffer with EGTA and EDTA (Boston Bioproducts, Ashland, MA) with protease inhibitor cocktail (P8340, Sigma-Aldrich). Conditioned media was collected from serum-free cell cultures, cells removed by centrifugation at 200 × g and protein concentrated using Amicon Ultra-15 3 kDa Centrifugal Filter Units (Millipore) at 3000 × g. Protein concentration was measured using a BCA (bicinchoninic acid) assay kit (Pierce/Thermo Scientific). Reduced protein in Laemmli sample buffer was resolved using SDS-PAGE and transferred to Immobilon-P 0.45 μm PVDF membrane (EMD Millipore, Billerica, MA). Membranes were blocked with 5% non-fat dry milk in PBS, incubated with primary antibody, followed by the appropriate secondary IgG antibody; sheep anti-mouse IgG HRP or donkey anti-rabbit IgG HRP linked (GE Healthcare). Membranes were washed thoroughly between steps using PBS containing 0.05% Tween-20, and developed using ECL Plus western blotting detection kit (GE Healthcare). Primary antibodies used for western blot analysis were as follows: EpCAM (C10, sc-25308), Integrin β4 (H-101, sc-9090), uPA (H-140, sc-14019) from Santa Cruz Biotechnology; AKT (#9272), p-AKT (#9271), S6K (#9202), p-S6K (#9205) from Cell Signaling. GAPDH (6C5) antibody was obtained from Abcam. Membranes were stripped using ReBlot Plus Strong Antibody stripping solution (EMD Millipore) before reprobing.

### Immunohistochemistry

Paraffin-embedded tumor tissue and lymph nodes were dewaxed, rehydrated, and stained with hematoxylin and eosin (H&E) or immunostained to detect human cytokeratin-18 (K18, Epitomics), EpCAM (Santa Cruz), E-Cadherin (BD Bioscience), mouse blood vessels (CD31, Pharmingen), or mouse lymphatic vessels (podoplanin, Reliatech). Antigen retrieval was performed with boiling citrate buffer (pH 6) for K18, EpCAM and E-cadherin or with proteinase K for podoplanin and CD31. Endogenous peroxidases were blocked with 3% peroxide in methanol. Tissues were blocked using normal serum and incubated with primary antibodies overnight at 4°C, biotinylated secondary antibodies (Vector Laboratories, Burlingame, CA) for one hour, and Vectastain Elite (avidin-HRP; Vector) for 30 min, and finally developed with diaminobenzidine chromogen (DAB, Vector). To detect human epithelial cell metastases, sentinel lymph node sections were stained with K18, counterstained with hematoxylin, examined by microscopy and K18-positive cells in small foci were scored as metastases. Single K18-positive cells in the lymph node were not scored as metastases. Three different tissue levels from each of two lymph nodes (when available) were examined per mouse.

### *In vivo* tumor experiments

Eight week old male Balb/c *Nu/Nu* mice were purchased from Massachusetts General Hospital and housed in the Animal Resource at Children’s Hospital (ARCH) facility accredited by the American Association for Accreditation of Laboratory Animal Care (AAALAC). All experiments were conducted in accordance with the principles and procedures outlined in the NIH Guide for the Care and Use of Laboratory Animals and approved by an Institutional Animal Care and Use Committee (IACUC) at Boston Children’s Hospital. For orthotopic prostate injections, mice were anesthetized and an abdominal incision was made to expose the prostate. 2×10^6^ cells ( suspended in 40 μl HBSS) were injected into the prostate using a Hamilton mini-injector, and the incision was closed with 9 mm wound clips. Tumor growth was monitored by palpation. After 4–12 weeks (5 weeks for direct comparison experiment), mice were sacrificed and necropsied. Tumors (and lymph nodes in 5 wk experiment) were removed, weighed and measured with calipers, fixed in formalin and processed for paraffin blocks. Orthotopic tumor volumes were calculated as widthSuperscript> × /Superscript> × length × 0.5. Sentinel paraaortic lymph nodes were washed with PBS, filtered through a 100 μm cell strainer (BD Biosciences), and plated in complete media on tissue culture dishes. The following day, cells were washed thoroughly with PBS, replaced with fresh complete media and re-named DU145-LN1 (from lymph node). After expansion in culture, in vivo orthotopic prostate injection was repeated for additional rounds of selection with subsequent cells named DU145-LN2, then DU145-LN3, and finally DU145-LN4.

For skin tumors, 5×10^6^ cells were injected subcutaneously into the right dorsal flank of 8 week old male Balb/c *Nu/Nu* mice. Tumor size was measured externally with calipers, and tumor volume was calculated as V = widthSuperscript> × /Superscript> × length × 0.5.

### Gene expression analysis

RNA for cDNA microarray analysis was purified using RNeasy mini kits (Qiagen). Purity and integrity was confirmed by spectrophotometer and agarose gel. Total RNA was labeled and amplified according to manufacturer’s instructions by the Microarray Core Facility of the Molecular Genetics Core Facility at Boston Children’s Hospital supported by NIH-P50-NS40828 and NIH-P30-HD18655. DU145, DU145-LN1, DU145-LN2 and DU145-LN4 RNA samples were run on Illumina HumanRef-8 BeadChips (Illumina, San Diego, CA). Raw data were analyzed in BRB-ArrayTools (Biometric Research Branch, National Cancer Institute, Bethesda, MD, USA, http://linus.nci.nih.gov/BRB-ArrayTools.html).

Signal intensity data was subject to rank invariant normalization. Duplicated probes on the array were treated independently during normalization and statistical analyses. Negative or low intensity signals <10 were corrected to 10 to prevent extreme fold change artifacts.

Samples were subject to Hierarchical cluster analysis using Euclidian distance. Differentially expressed genes were identified using a time course analysis (DU145 as time = 0, and DU145LN1, LN2 and LN4 as time = 1, 2 and 3 respectively), with a cut-off minimum of 1.5-fold change in DU145-LN4 relative to DU145. For functional gene analysis, the entire dataset was imported into Ingenuity IPA Network Analysis software (Ingenuity Systems, Redwood City, CA), and we selected Cancer and Cellular Movement categories for further analysis. Cluster analysis of the relationship between cell types within these categories or of the entire gene probe population using one minus Pearson correlation, produced essentially indistinguishable dendrograms. We cross-referenced back to probe intensity values and genes were removed if all data points had low intensities of <100 Arbitrary Intensity Units. Selected genes were represented by heat map using GENE-E software (http://www.broadinstitute.org/cancer/software/GENE-E). For analysis of Cell Signaling, data were excluded if Illumina probe values were negative, <10, or less than the probe signal in the control group (DU145).

### Statistical analyses

Data from cell proliferation, migration and invasion assays were analyzed using unpaired two-sample student’s t-test. Statistical significance was considered at p ≤ 0.05. Specific p-values for each experiment are indicated in Figure Legends.

## Results

### Development and characterization of a new prostate cancer metastasis model *in vivo*

**Figure 1:**
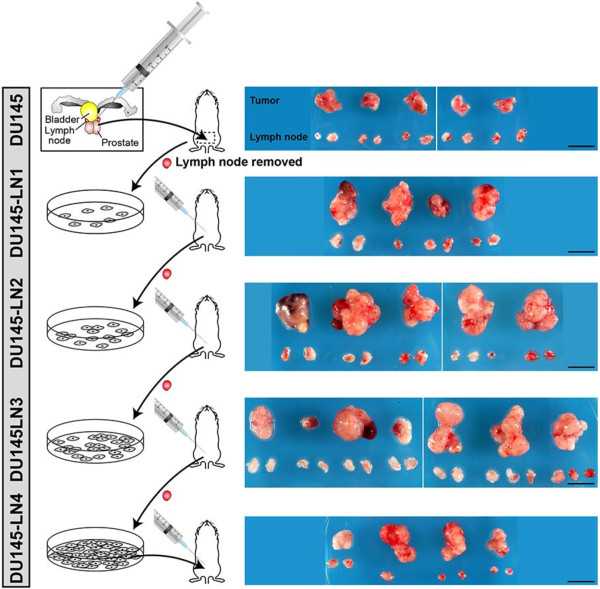
**Development of a model of metastatic prostate cancer through repeated selection of spontaneous lymph node metastases from orthotopic DU145 human prostate tumors.** Schematic (left panels) of the experimental approach shows orthotopic prostate inoculation of DU145 cells. Lymph nodes were removed and cultured, and selected tumor cells subject to repeated rounds of orthotopic injection. Right panels show gross anatomy of tumors and lymph nodes 5 weeks after all DU145 sublines were reinjected (this figure is modified with permission from [21]). Scale bar = 1 cm.

In order to select for prostate cancer cells with increased metastatic potential we used an *in vivo* cycling approach [12,18]. The DU145 human prostate cancer cell line [20] was used to establish a series of metastatic variants. DU145 cells were injected orthotopically into the prostate of immunodeficient *Nu/Nu* (nude) male mice. After the tumor was palpable (4–8 weeks), mice were euthanized and the sentinel paraaortic lymph nodes were removed and minced sterilely, and the cells placed into culture as described in Methods (Figure 1 left side). If no tumor cell outgrowth occurred, tumors in the remaining mice were allowed to grow for additional 2 week intervals before lymph nodes were removed and a cell line was established. Cells, now called DU145-LN1, were expanded in culture for several passages to eliminate fibroblast contamination, and re-injected orthotopically into the ventral lobes of the prostate of subsequent nude male mice. Repeated rounds of *in vivo* cycling were performed to establish the DU145-LN2, DU145-LN3 and DU145-LN4 cell lines. All cell lines were analyzed by RT-PCR for mouse and human GAPDH expression to ensure that only human cells and no mouse stromal cells were injected [21].

**Figure 2:**
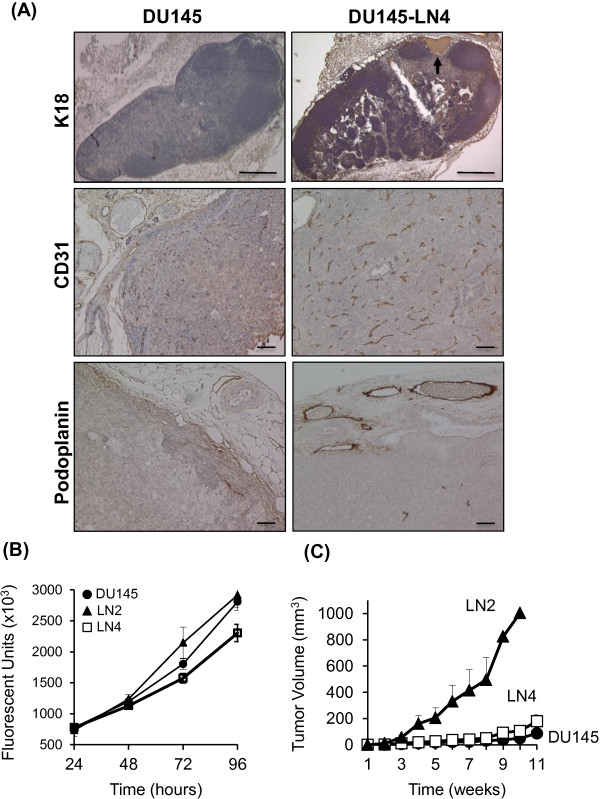
**DU145 LN tumors show increased growth, angiogenesis, lymphangiogenesis, and metastasis. (A)** Metastasis was measured by K18 staining of lymph nodes from mice bearing DU145 tumor (top left panel) and DU145-LN4 tumor (top right panel). K18-positive (brown color) tumor foci (arrow) were counted as positive incidence of metastasis. Scale bars = 0.5 mm. Tumor vascularization was assessed by CD31 IHC (brown color) of DU145 (middle left panel) and DU145-LN4 (middle right panel) prostate tumors. Increased vascularization was observed in DU145-LN4 tumors relative to DU145 tumors. Scale bars = 100 μm. Lymphangiogenesis was measured by podoplanin staining. Enlarged podoplanin-positive vessels (brown color) were observed in DU145-LN4 orthotopic tumors (lower right panel), compared to DU145 tumors (lower left panel). All sections were counterstained with hematoxylin (blue color). Scale bars = 100 μm. **(B) ***In vitro* proliferation assays of the DU145LN sublines indicate similar proliferation rates with slight reduced proliferation of DU145-LN4. 2.5x10^3^ cells plated/well, absorbance measured using Cyquant dye (Ex = 485 nm). Data in arbitrary fluorescence units x1000. Filled circles: parental DU145, filled triangles: DU145-LN2, empty squares: DU145-LN4. Error bars indicate S.D. of triplicate wells. **(C)** DU145-LN2 shows increased tumor growth compared to parental DU145 when injected subcutaneously into nude mice. 5x10^6^ cells were injected into the flank. Symbols as above.

Once all cell lines were established, our *in vivo* metastatic model was tested and characterized by injecting each cell line orthotopically into the prostate of mice simultaneously (n = 4-7 mice per group). Tumors and lymph nodes were removed after 5 weeks, as shown in Figure 1 (right side). Tumor incidence was 100% in all groups, but local metastatic incidence varied (Table 1). To quantify metastases, paraffin-embedded lymph node sections (3 levels per lymph node, 4–5 mice per group) were analyzed by H&E and human K18 immunostaining. Lymph node metastasis was recorded as incidence of K18-positive foci per mouse. Repeated rounds of metastatic selection increased the incidence of K18-positive metastatic foci from 0% in parental DU145 lymph nodes, to ≥75% in DU145-LN2, DU145-LN3 and DU145-LN4 lymph nodes (Figure 2A, Table 1). In addition to enhancing the metastatic potential of the DU145-LN sublines, our *in vivo* cycling approach also increased the growth of these tumors. Orthotopic prostate tumor size was significantly increased from DU145 to DU145-LN1 and further to DU145-LN2 (Figure 1 right side, Table 1). Interestingly, we found that DU145-LN2 was consistently the largest tumor when injected into the prostate, with rapid tumor growth compared to that of the DU145 parental cell line. While increased tumorigenic and metastatic ability appeared to have been established by the LN2 generation, we also employed DU145-LN4 cells in many of our studies as we surmised it was likely to represent the most stable and homogenous cell line.

**Table 1:** ***In vivo *** **orthotopic growth and metastasis of DU145 sublines**

**Cell line injected**	**% Tumor incidence**	**Mean tumor weight (g) (±SD)**	**Mean tumor diameter (mm) (range)**	% Metastatic incidence*
DU145	100	0.66 (0.09)	7.3 (6.3-9.3)	0
DU145-LN1	100	0.99 (0.25)	11.9 (8.3-14.1)	50
DU145-LN2	100	1.80 (0.37)	15.8 (12.9-18.2)	75
DU145-LN3	100	1.16 (0.45)	12.1 (5.5-16.1)	83
DU145-LN4	100	n.d.	10.2 (7.3-12.8)	75

Cells were injected into the prostate. After 5 weeks, tumors and lymph nodes were removed. *K18-positive metastatic foci in lymph nodes. All available lymph nodes were evaluated.

The increase in tumor size in the DU145-LN model was not explained by changes in the cell proliferation rate *in vitro*. The proliferation rate of DU145-LN2 cells was not significantly different than parental DU145 cells, while DU145-LN4 consistently showed a slightly reduced proliferation rate *in vitro* (Figure 2B). We further investigated tumor growth potential by assessing subcutaneous tumor growth over time. Subcutaneous tumor size is more accurate and straightforward to measure compared to intraprostatic tumor size. Tumor cells were injected subcutaneously into the dorsal right flank of nude male mice, and tumor size was measured externally with calipers. DU145 cells were less tumorigenic when injected into the skin of nude mice. In independent experiments, we found that an average of 7/10 mice that received DU145 cells showed tumor-take. To our surprise, DU145-LN2 cells were highly proliferative when injected subcutaneously, relative to parental DU145 control cells (Figure 2C). DU145-LN2 cells showed tumor take in 9/10 and 10/10 mice, more rapid tumor take and more rapid tumor growth. DU145-LN4 cells injected into the skin showed tumor take in 10/10 mice and similar growth rate relative to parental DU145 cells (Figure 2C). Metastatic potential was not evaluated in the ectopic experiments.The increase in lymph node metastasis and tumor size in our DU145-LN model was accompanied by greater vessel density in the DU145-LN4 as compared to DU145 prostate tumors, as observed by CD31 immunostaining (Figure 2A, middle panels). Despite remaining relatively small, DU145 tumors were often observed to be necrotic in their center (Figure 2A middle left). This observation is likely related to their low recruitment of supporting CD31-positive blood vessels. Since we “selected” for metastasis to regional lymph nodes in our model, we anticipated that these cells would use lymphatic vessels as a conduit. In fact, LVI and lymphangiogenesis can predict metastatic potential. The more aggressive tumors DU145-LN2 (not shown) and DU145-LN4 showed more numerous, enlarged peri-tumoral lymphatic vessels (as detected by podoplanin staining) compared to DU145 tumors, indicating increased lymphangiogenesis (Figure 2A, lower panels).

### Metastatic selection changes prostate tumor cell phenotype

**Figure 3:**
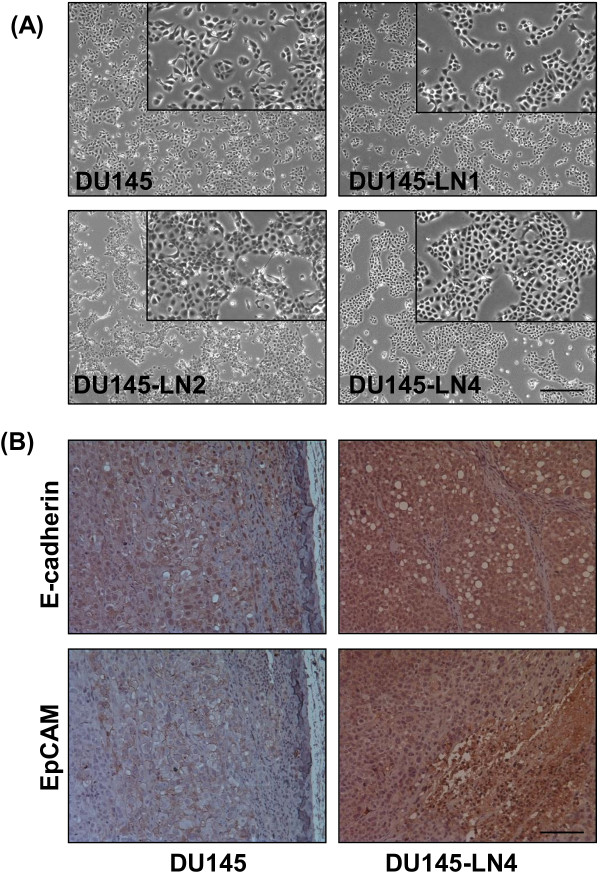
**Phenotype of DU145-LN cells with increased metastatic ability. (A)** Phase contrast microscopy images of parental DU145 cells, DU145-LN1, DU145-LN2, and DU145-LN4 cells. Cells exhibit progressive phenotypic changes after selection, with increased clustering and cell-cell adhesions from parental DU145 (top left panel) to DU145-LN4 (bottom right panel). Insets are larger images of lower panels. Scale bar = 0.5 mm **(B)** DU145-LN4 tumor cells maintain their mesenchymal-epithelial transition (MET) phenotype *in vivo*. IHC of DU145 and DU145-LN4 subcutaneous tumor tissue with the epithelial markers, E-cadherin and EpCAM. High expression of E-cadherin and EpCAM was maintained in the tumor tissue. Scale bar = 100 μm.

The selection of DU145 metastatic variants resulted in a progressive change in cell phenotype. DU145 human prostate tumor cells are a heterogeneous epithelial cell population in *in vitro* culture [22]. We recently showed that the more metastatic DU145-LN cells undergo mesenchymal to epithelial transition (MET)-like changes in gene expression [21]. As seen in phase contrast microscopy, metastatic DU145-LN4 cells form more clusters *in vitro*, with more cell-cell interactions than DU145 cells (Figure 3A). The intermediate DU145-LN cell lines displayed intermediate phenotypes. These expression changes were maintained in *in vivo* tumors. Immunohistochemical staining of paraffin embedded subcutaneous tumors showed higher expression of E-cadherin and EpCAM by DU145-LN4 compared to DU145 (Figure 3B).

### Increased migration and invasion in metastatic DU145-LN cells

**Figure 4:**
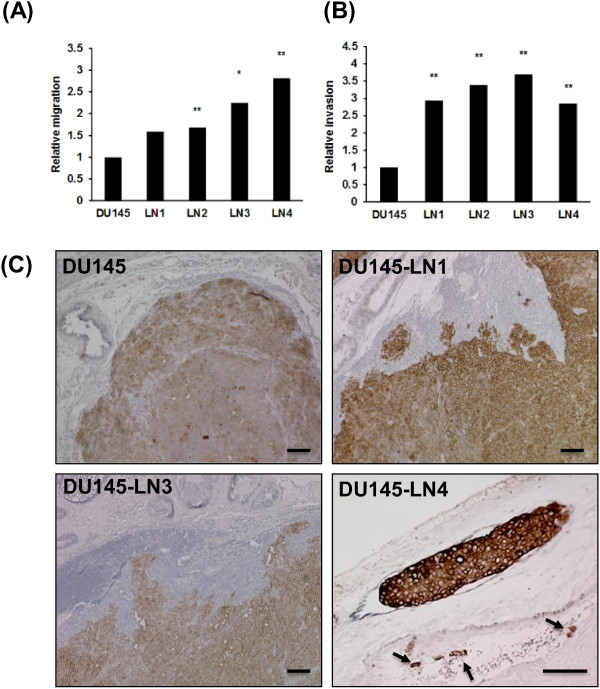
**DU145-LN sublines exhibit increased migration and invasion with metastatic ability, relative to DU145 cells.** DU145-LN metastatic sublines show increased **(A)** cell migration in the transwell migration assay and **(B)** increased invasion in the Matrigel invasion assay. For both assays: Mean of triplicate assays ± S.D. Student t-test, *p < 0.05, **p < 0.01, **(C)** IHC of orthotopic prostate tumors with K18 (brown) and counterstained with hematoxylin (blue). Margins were smooth and well-defined in the DU145 tumors (top left panel), while invasive margins were observed in DU145-LN1 (top right panel) and DU145-LN3 (lower left panel) tumors. Right lower panel shows magnified image of DU145-LN4 tumor seen in Figure 2A. Double staining of K18 (brown) and podoplanin (black) shows tumor foci present in an enlarged lymphatic vessel and in a tumor-associated blood vessel (arrows) (lower right panel). Scale bars = 100 μm.

To further characterize our new prostate cancer model, we examined the effect of this metastatic selection on cell behavior *in vitro*. Cell migration and invasion are important steps in the process of metastasis [23]. The effect of metastatic selection on DU145 cell migration was determined in the transwell migration assay. 1% fetal bovine serum (FBS) was used in the lower wells as a chemoattractant, and cells were allowed to migrate for 4 hours. We found that there was a progressive increase in cell migration from the DU145 parental cell line to the metastatic DU145-LN4 cells. Parental DU145 exhibited a low level of migration toward 1% FBS on collagen-coated membranes, while metastatic DU145-LN4 cells displayed over 2.5-fold higher migration (Figure 4A). The migration of DU145-LN2, LN-3 and LN-4 was significantly greater than DU145.We next looked at the invasive behavior of the DU145-LN cells using the transwell Matrigel invasion assay. In this assay, cells are required to invade through an extracellular matrix barrier. 5% FBS was used as a chemoattractant and cell invasion was assessed after 12 hours. The metastatic DU145-LN sublines also showed significantly increased invasive abilities compared to parental DU145 cells. Figure 4B demonstrates that DU145-LN1 to LN4 all showed over 2.5 fold higher invasion, compared to parental DU145 cells.

The increased invasion of DU145-LN1-4 cells was also observed *in vivo*. Orthotopic prostate tumor tissue was stained with human K18 to visualize tumor margins. DU145 tumor margins were largely defined and well circumscribed (Figure 4C top left). DU145-LN subline tumors showed highly invasive edges with human K18-positive tumor cells protruding into the mouse prostate gland (Figure 4C top right, bottom left). Interestingly, in DU145-LN4 tumors K18-positive tumor emboli were also clearly visible inside lymphatic vessels in the peritumoral stroma, as visualized by double staining with podoplanin (black color) and K18 (brown color) (Figure 4C). Tumor cells were also seen inside tumor-associated blood vessels (Figure 4C bottom right, arrows).

### Identification and analysis of genes involved in cell invasion and migration

**Figure 5:**
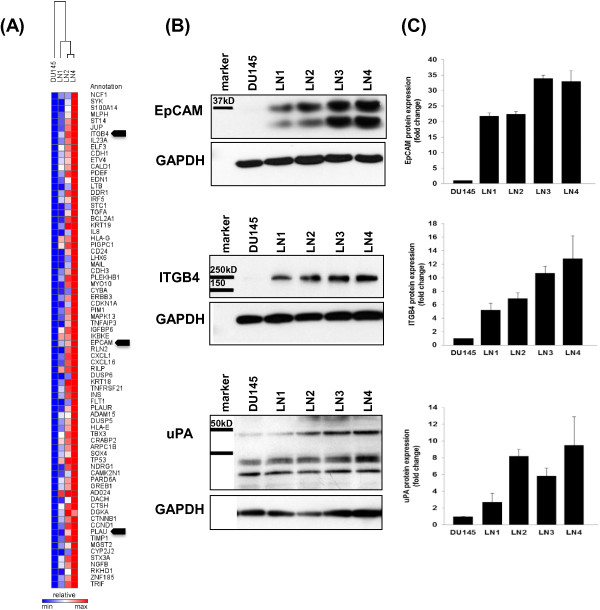
**Gene expression analysis in the DU145-LN metastatic sublines and validation of increased EpCAM, integrin β4 and uPA protein expression. (A)** Heat map of cell-movement and cancer genes increased in the more metastatic DU145-LN sublines, relative to DU145 cells. Relative expression color scale from high (red) to low (blue). Arrowheads indicate genes selected for further analysis. **(B)** Western blot analysis of EpCAM, integrin β4 and uPA expression in whole cell lysate showed progressive increase in expression in the DU145-LN metastatic sublines. 20 μg protein was loaded to detect EpCAM, 70 μg protein was used for integrin β4 and uPA western blots. GAPDH shown as protein loading controls. **(C)** Band intensity for each protein was evaluated using ImageJ software and averaged from 2–4 western blots each.

To investigate the molecular changes underlying the gain of metastatic potential in our new model, we analyzed the gene expression profile of these cells using an Illumina cDNA Ref6 bead expression array. RNA was isolated from these cells between passage 6–8. We confirmed through RT-PCR that cell cultures were not contaminated with cells of mouse origin that might share identity in gene probe sequence [21]. Gene expression data was normalized as described in Methods. Hierarchical Cluster analysis confirmed that DU145-LN1 was most closely related to the parental DU145 cells in gene expression profile. The more metastatic cells, DU145-LN2 and DU145-LN4, clustered together and were progressively more divergent from DU145 parental cells, as visualized by dendrogram (Figure 5A).

**Figure 6:**
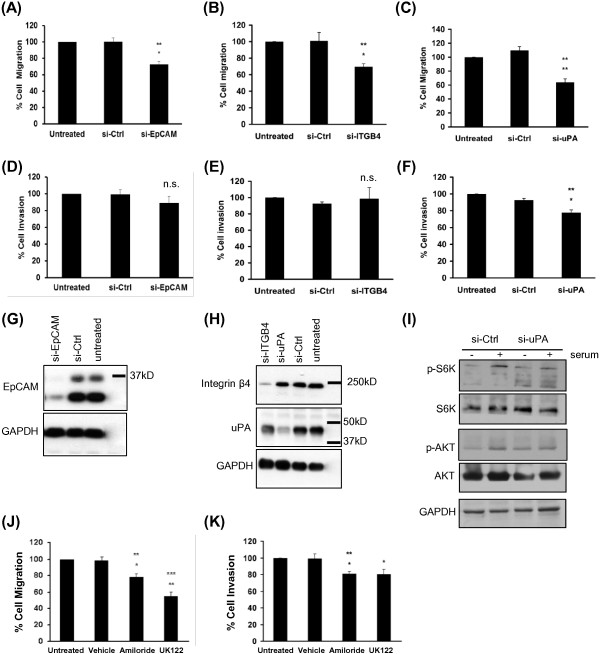
**The role of EpCAM, integrin β4 and uPA in migration and invasion of DU145-LN4 cells.** Cell migration of DU145-LN4 cells in a transwell migration assay **(A-C)** and a Matrigel invasion assay **(D-F)**. siRNA knockdown of **(A)** EpCAM, **(B)** integrin β4, or **(C)** uPA significantly inhibited cell migration relative to control siRNA and untreated cells. siRNA knockdown of **(D)** EpCAM or **(E)** ITGB4 did not significantly affect cell invasion in a Matrigel invasion assay. **(F)** uPA siRNA significantly inhibited cell invasion relative to control siRNA and untreated cells. Western blot analysis of whole cell lysates from cells treated with siRNA in parallel with migration/invasion assays: **(G)** EpCAM, **(H)** integrin β4, and uPA western blots. Blots were probed with GAPDH as loading controls. **(I)** Western blot analysis of whole cell lysates from cells treated with control siRNA or si-uPA and serum starved (−) or serum stimulated (+). p-AKT and p-S6K were induced after serum addition in control cells but not in cells lacking uPA. Total AKT, S6K, and GAPDH served as controls. DU145-LN4 cell migration **(J)**, and cell invasion **(K)** were significantly inhibited by treatment with 10 μM amiloride or 10 μM UK122. Students t-test, *p ≤ 0.05, **p ≤ 0.01, n.s.-not significant. Top asterisked p value represents analysis between untreated cells and specific siRNA, lower value indicates analysis between control siRNA treated cells and specific siRNA.

To identify genes related to metastasis we applied a continuous scale time course analysis using BRB Array Tools. Our analysis revealed a pattern of gene expression changes that showed progressively increased or decreased expression across the cell lines, from parental DU145 cells to DU145-LN2 or DU145-LN4 cells. These gene expression changes correlated with the increased migration, invasion and metastatic potential of the cell lines. We used Ingenuity software analysis to select for genes upregulated in cancer and cellular movement, as described in Methods. Figure 5A shows a heat map generated using cancer and cell movement genes significantly increased (red color) in DU145-LN4 cells. The genes included ITGB4 (integrin β4), ST14 (Matriptase), EPCAM (Epithelial Cell Adhesion Molecule, (EpCAM)), CDH1 (E-cadherin), JUP (junction plakoglobin/desmoplakin 3/γ-catenin) and PLAU (urokinase plasminogen activator (uPA)). Three of these genes were further analyzed in this study: Epithelial Cell Adhesion Molecule (EpCAM), urokinase plasminogen activator, (uPA, gene name PLAU), and integrin β4 (ITGB4). Relative cDNA expression levels are shown in Additional file 1: Table S1. In addition, Ingenuity software was used to select for cellular signaling genes differentially regulated among the cell lines (Additional file 1: Figure S1).We investigated whether protein expression levels correlated to the RNA expression profiles using immunoblotting of whole cell lysates. EpCAM, integrin β4 and uPA all showed a progressive increase in protein expression from parental DU145 to DU145-LN4 cells (Figure 5B). GAPDH immunoblotting was used to confirm equal protein loading. ImageJ software was used to measure protein band intensity and averaged from 2–4 western blots for each protein and graphed in Figure 5C. The protein expression levels showed good correlation with the microarray data for these selected genes.We investigated whether these proteins were involved in the increased migration observed in the metastatic DU145-LN cells. DU145-LN4 cells were transfected with siRNA against EpCAM, uPA or integrin β4, and the effect on cell migration was measured in the transwell assay after 48 hours. Our data show that siRNA knockdown of either EpCAM, integrin β4 or uPA using siRNA significantly inhibited cell migration (Figure 6A-C). We also examined whether silencing these genes would affect cell invasion. uPA silencing significantly inhibited tumor cell invasion, while both EpCAM or integrin β4 knockdown had no significant effect on DU145-LN4 cell invasion (Figure 6D-F). Whole cell lysates were collected in parallel and effective protein knockdown by siRNA treatment was confirmed by immunoblotting (Figure 6G-H). In addition, two chemical inhibitors of uPA were able to inhibit cell migration and invasion of DU145-LN4 cells (Figure 6J-K, respectively). Downstream cell signaling pathways were evaluated following uPA knockdown in DU145-LN4 cells. Specifically, phosphorylation of AKT and phosphorylation of p70 S6 kinase (S6K) were upregulated in control cells following serum stimulation, but these proteins were not activated in uPA-lacking cells (Figure 6I).

In summary, we have established a new model of human prostate cancer metastasis using DU145 cells, a widely used androgen-independent human prostate cancer cell line. This model represents an advance upon other widely available prostate metastasis cell models, because the intermediate cell lines are available for analysis. Our selection approach has produced more highly metastatic, migratory and invasive sublines. Our initial analysis has revealed a subset of genes that correlate with metastatic potential. Cell migration and invasion are important steps in metastasis; we have shown that three molecules upregulated in our model: EpCAM, integrin-β4 and uPA play roles in these processes.

## Discussion

Our goal was to create a new reliable human prostate cancer model system that would use human prostate tumor cells and result in rapidly growing (non-necrotic) tumors in 100% of the mice injected and consistently recapitulate the invasive and metastatic properties seen in patients. Many prostate cancer research studies use one of three human cell lines: PC3, LNCaP or DU145. While each cell line has its benefits and drawbacks, we focused on the DU145 cell line since it did not have well-used metastatic sublines reported in the literature. In our search for metastasis-related pathways, we had also wanted to select an androgen independent cell line. These cells grow robustly *in vitro* and express many prostate and epithelial markers, yet they grow poorly in mice even when injected into the mouse prostate gland. Therefore, many labs resort to injecting high numbers (>2×10^6^) of cells and co-injecting ECM components or fibroblasts to enhance tumor-take and angiogenic potential. We chose to select for highly metastatic variants of DU145 using an *in vivo* cycling strategy that was previously successful for PC-3 M and LNCaP [12,18].

Herein, we have presented the establishment and characterization of our new model of human prostate cancer, the DU145-LN metastatic series. The DU145-LN cells show enhanced *in vivo* growth as well as migratory, invasive and metastatic abilities. These cell lines represent new tools to explore the process of metastasis. Our approach to developing the DU145-LN metastatic series via spontaneous metastasis from orthotopic organ sites provided the tumor with appropriate micro-environmental signals [14]. Cells with the ability to spontaneously metastasize from the prostate tumor and survive in the lymph node were repeatedly selected. The role of lymphatic versus hematologic metastasis has been debated [24,25]. However, the presence of tumor-positive lymph nodes continues to be an important predictor of distant metastases and patient survival in many cancers [9,26]. Studies have shown that lymphovascular invasion is significantly associated with PSA biochemical recurrence and patient survival in prostate cancer [5,27], although LVI may not significantly improve predictive accuracy above standard clinicopathological features in prostate cancer [11]. We clearly observed tumor foci in the enlarged lymphatics of the DU145-LN4 orthotopic tumors, indicating that our model recapitulates steps common in human prostate cancer progression.

After completing four rounds of cycling the DU145 cells in mice (prostate to lymph node), we compared all five cell lines in a head-to-head comparison for tumorigenicity and metastatic potential in a 5 week period (Figure 1 shows gross images of resulting tumors). DU145 was poorly angiogenic and had a low vascular density, therefore resulting in small tumors. Cycled tumors had higher microvessel densities, and vessel density has been shown to correlate with metastatic potential in human prostate cancer [28]. In addition, the DU145-LN4 tumors had increased lymphangiogenesis surrounding the tumors and invasive leading edges. Lymphangiogenesis has been shown to be an important mechanism of prostate cancer metastasis [26,29], and has been our focus in this study.

Most human prostate cancer cells do not grow well subcutaneously; however, our new DU145-LN2 cell line represents a useful and rapid non-surgical xenograft model for tumor growth studies in the skin, e.g. drug screening. Metastatic cycling of DU145 prostate cancer cells also resulted in cells that were more motile and invasive. By examining the gene expression profiles of these cells we revealed many genes correlating with their metastatic ability. In this report we have demonstrated the involvement of EpCAM, integrin β4 and uPA in tumor cell migration and/or invasion--key steps in the metastatic cascade. Although each of these genes may not be individually competent to induce metastasis in parental cells, we propose that our model represents a valuable and relevant system, as the genes we have identified have been shown to be clinically important in prostate cancer.

EpCAM (also known as CD326) is well established as a tumor marker in many carcinomas, and is widely used to purify circulating tumor cells from blood [30]. EpCAM is a transmembrane glycoprotein and has diverse functions in cell-cell adhesion, migration, proliferation and differentiation [31]. In human prostate cancer, several tissue studies have shown upregulated EpCAM in the tumor epithelium and in metastatic lesions [32-35]. EpCAM expression in prostate tumor tissue is also a significant predictor of shorter biochemical recurrence free-survival [35]. The mechanism of EpCAM activity has not yet been well defined. EpCAM can be cleaved in its ectodomain to release an extracellular fragment, (EpEX) and this may affect E-cadherin mediated cell-cell adhesion [36]. It is possible that this fragment may be involved in the increased migration and invasion observed in the DU145-LN4 cells. High expression of the epithelial marker E-cadherin has been associated with stronger cell-cell interaction and subsequent reduced cell motility [36]. However, the presence of EpEX may modulate this role. Our Western blot analysis (Figure 5B) indicates that both full length EpCAM and EpEX is present at high levels in the DU145-LN4 (and DU145-LN2) cells. EpCAM also associates with the tight junction protein, claudin 7, to promote tumor cell migration rather than cell-cell adhesion that leads to lymphatic spread [37]. Claudin 7 expression was also dramatically upregulated in the DU145-LN cell series in microarray data (relative to DU145, DU145-LN1 had 9.6X, DU145-LN2 had 21X and DU145-LN4 had 28X fold higher claudin-7 expression). Antisense knockdown of either EpCAM or claudin-7 reduces tumor growth and metastasis in mice, and knockdown of both is more effective [38].

The cell surface EpCAM complexes can also involve an additional partner identified in our gene expression analysis, β4 integrin. In normal epithelial cells α_6_β_4_ resides in hemidesmosomes. In tumor cells, integrin β4 can relocate from hemiodesmosomes to the leading edge of migrating cells where it is involved in the signaling of many receptor tyrosine kinases, including ErbB2, ErbB3, EGFR and Met [39-41]. β4 integrin therefore impacts cell signaling, migration and invasion through multiple pathways. β4 expression also influences multiple miRNAs impacting cell motility [42]. High levels of β4-integrin have been found across many prostate cancer tissue expression studies, and in metastatic and castrate-resistant prostate cancer metastases [41]. Transgenic mice with a β4-integrin signaling domain *mutation* showed reduced prostate tumor formation and progression, thus supporting our data that ITGB4 is involved in tumor cell migration and metastasis [41].

We also showed that uPA expression positively correlated with metastatic potential in the DU145-LN cell series. uPA silencing significantly inhibited both tumor cell migration and invasion. Serine proteases, such as uPA play an important role in tumor progression. By degrading the extracellular matrix and basement membrane they can promote cell invasion, angiogenesis and metastasis [43]. Circulating levels of uPA, and its receptor uPAR (urokinase-type plasminogen activator receptor), are significantly elevated in prostate cancer patients, and are higher in patients with lymph node and bone metastases, compared to those with non-metastatic disease [44,45]. uPA and uPAR levels correlate with Gleason score, extracapsular extension, LVI, seminal vesicle and lymph node invasion and are also associated with biochemical progression and poor prognosis [45,46]. Both uPA and uPAR are involved in Matrigel invasion in PC-3 cells [47,48], and RNAi or shRNA knockdown of uPA and uPAR reduced orthotopic prostate tumor size via apoptosis. In DU145 cells, uPAR over-expression increased Matrigel invasion *in vitro* which was inhibited by uPA antibody or inhibitor. In addition, stable overexpression of uPAR was accompanied by uPA upregulation [49], providing additional evidence for the interdependence of the protease and receptor activities.

The uPA protease axis appears to play an important role in the invasive and metastatic behavior of our metastatic model. The uPA receptor, uPAR (gene name PLAUR) also showed increased gene expression as DU145 cells become more metastatic; with 1.3X fold increased expression in LN1, 2.0X fold in LN2, and 2.7X fold in LN4, relative to parental DU145 cells. Furthermore, one of the key activators of uPA activity is the protease Matriptase (gene name ST14). Matriptase was also highly upregulated in our model of prostate cancer metastasis; 4.5X fold in DU145-LN1, 11X fold in DU145-LN2 and 16X fold increased in DU145-LN4, relative to DU145 (Figure 5A). Antagonists of the uPA/uPAR axis have been suggested for use as anti-tumor agents with targeted monoclonal antibodies and nanoparticles currently under development [50].

Our model has identified a network of gene and pathway changes spontaneously arising as cells became more metastatic. These include EpCAM, β4-integrin and uPA. Clearly many of these pathways may interact and feedback upon each other. There may be master regulators in this system, such as transcription factors and/or microRNAs that influence expression of these and many other genes. Indeed, ZEB1 has been reported to regulate EpCAM, β4-integrin and uPA [51-53]. In turn, the miR-200 family regulates the epithelial phenotype and ZEB1 [54-56]. In addition, there are other transcription factors related to cancer and cell movement that are significantly upregulated in this model, including ELF3 (8.7X higher in DU145-LN4) and ETV4 (7.5X fold higher level in DU145-LN4 compared to DU145 cells). These and other genes may present new targets for intervention in metastatic cell behavior.

## Conclusions

Using one of the “classical” human prostate cancer cell lines, DU145, we have developed a series of new metastatic variants, DU145-LN1 to DU145-LN4, through *in vivo* cycling of spontaneous lymph node metastases. The metastatic cells are more migratory and invasive. Gene expression analysis revealed many genes correlating with metastatic ability. We show that EpCAM and integrin β4 are involved in migration, while uPA is involved in migration and invasion of metastatic prostate cancer cells.

Our analysis of the role of these genes has demonstrated the relevance of our new system. We expect that further study of downregulated genes and as yet uncharacterized cDNAs with strong correlation to metastatic ability will bring new discoveries. We propose that our new model system will be a powerful and additional tool to interrogate the metastatic cascade in prostate cancer.

## Abbreviations

EpCAM: Epithelial cell adhesion molecule; ITGB4: Integrin β4; uPA: Urokinase plasminogen activator; uPAR: Urokinase plasminogen activator receptor; siRNA: Small interfering RNA; IHC: Immunohistochemistry; FBS: Fetal bovine serum; K18: Cytokeratin 18; MET: Mesenchymal to epithelial transition.

## Competing interests

The authors declare that they have no competing interests.

## Authors’ contributions

JB conceived of the study, developed the animal model and cell lines, performed microarray analyses and wrote the manuscript; IC participated in *in vivo* experiments and edited the manuscript; MM performed immunohistochemistry; DTP carried out the migration, invasion and western blotting; AMW performed the proliferation assay, immunohistochemistry and participated in subcutaneous tumor experiments. BRZ participated in study design and edited the manuscript. DRB conceived of the study, developed the animal model and cell lines, performed immunohistochemistry and western blotting, and edited the manuscript. All authors read and approved the final manuscript.

## Pre-publication history

The pre-publication history for this paper can be accessed here:

http://www.biomedcentral.com/1471-2407/14/387/prepub

## Supplementary Material

Additional file 1: Figure S1 Heat map using Ingenuity Analysis software and the “Cell Signaling” category. The range of difference between DU145 and DU145LN4 groups are 1.8-16.4 fold. **Table S1.** Relative expression levels of PLAU, EPCAM, ITGB4 and housekeeping genes in the metastatic DU145-LN sublines. Click here for file

## References

[B1] American Cancer Society Cancer Facts & Figures 2012 2012 Atlanta: American Cancer Society

[B2] Siegel R DeSantis C Virgo K Stein K Mariotto A Smith T Cooper D Gansler T Lerro C Fedewa S Lin C Leach C Cannady RS Cho H Scoppa S Hachey M Kirch R Jemal A Ward E Cancer treatment and survivorship statistics, 2012 CA Cancer J Clin 2012 62 4 220 241 10.3322/caac.21149 22700443

[B3] Smith JA Jr Seaman JP Gleidman JB Middleton RG Pelvic lymph node metastasis from prostatic cancer: influence of tumor grade and stage in 452 consecutive patients J Urol 1983 130 2 290 292 6876275 10.1016/s0022-5347(17)51112-x

[B4] Dadras SS Paul T Bertoncini J Brown LF Muzikansky A Jackson DG Ellwanger U Garbe C Mihm MC Detmar M Tumor lymphangiogenesis: a novel prognostic indicator for cutaneous melanoma metastasis and survival Am J Pathol 2003 162 6 1951 1960 10.1016/S0002-9440(10)64328-3 12759251 PMC1868148

[B5] Cheng L Jones TD Lin H Eble JN Zeng G Carr MD Koch MO Lymphovascular invasion is an independent prognostic factor in prostatic adenocarcinoma J Urol 2005 174 6 2181 2185 10.1097/01.ju.0000181215.41607.c3 16280760

[B6] Rinderknecht M Detmar M Tumor lymphangiogenesis and melanoma metastasis J Cell Physiol 2008 216 2 347 354 10.1002/jcp.21494 18481261

[B7] Karakiewicz PI Hutterer GC Predictive models and prostate cancer Nat Clin Pract Urol 2008 5 2 82 92 10.1038/ncpuro0972 18259186

[B8] Sleeman JP Thiele W Tumor metastasis and the lymphatic vasculature Int J Cancer 2009 125 12 2747 2756 10.1002/ijc.24702 19569051

[B9] Zwaans BM Bielenberg DR Potential therapeutic strategies for lymphatic metastasis Microvasc Res 2007 74 2–3 145 158 17950368 10.1016/j.mvr.2007.08.006 PMC2525453

[B10] Wilt TJ Brawer MK Jones KM Barry MJ Aronson WJ Fox S Gingrich JR Wei JT Gilhooly P Grob BM Nsouli I Iyer P Cartagena R Snider G Roehrborn C Sharifi R Blank W Pandya P Andriole GL Culkin D Wheeler T Prostate Cancer Intervention versus Observation Trial (PIVOT) Study Group Radical prostatectomy versus observation for localized prostate cancer N Engl J Med 2012 367 3 203 213 10.1056/NEJMoa1113162 22808955 PMC3429335

[B11] Aizer AA Paly JJ Zietman AL Nguyen PL Beard CJ Rao SK Kaplan ID Niemierko A Hirsch MS Wu CL Olumi AF Michaelson MD D'Amico AV Efstathiou JA Multidisciplinary care and pursuit of active surveillance in low-risk prostate cancer J Clin Oncol 2012 30 25 3071 3076 10.1200/JCO.2012.42.8466 22851571

[B12] Stephenson RA Dinney CP Gohji K Ordonez NG Killion JJ Fidler IJ Metastatic model for human prostate cancer using orthotopic implantation in nude mice J Natl Cancer Inst 1992 84 12 951 957 10.1093/jnci/84.12.951 1378502

[B13] Perrotte PJR Bielenberg DR Eve BY Dinney CPN Organ-specific angiogenesis and metastasis of human bladder carcinoma growing in athymic mice Mol Urol 1997 1 4 299 307

[B14] Bielenberg DR Fidler IJ Teicher BA Regulation of Angiogenesis by the Organ Microenvironment Antiangiogenic Agents in Cancer Therapy 1999 6 Totowa: Humana Press 77 91

[B15] Fidler IJ The organ microenvironment and cancer metastasis Differentiation 2002 70 9–10 498 505 12492492 10.1046/j.1432-0436.2002.700904.x

[B16] Hanahan D Weinberg RA Hallmarks of cancer: the next generation Cell 2011 144 5 646 674 10.1016/j.cell.2011.02.013 21376230

[B17] Sottnik JL Zhang J Macoska JA Keller ET The PCa tumor microenvironment Cancer Microenviron 2011 4 3 283 297 10.1007/s12307-011-0073-8 21728070 PMC3234329

[B18] Pettaway CA Pathak S Greene G Ramirez E Wilson MR Killion JJ Fidler IJ Selection of highly metastatic variants of different human prostatic carcinomas using orthotopic implantation in nude mice Clin Cancer Res 1996 2 9 1627 1636 9816342

[B19] Sobel RE Sadar MD Cell lines used in prostate cancer research: a compendium of old and new lines–part 1 J Urol 2005 173 2 342 359 10.1097/01.ju.0000141580.30910.57 15643172

[B20] Stone KR Mickey DD Wunderli H Mickey GH Paulson DF Isolation of a human prostate carcinoma cell line (DU 145) Int J Cancer 1978 21 3 274 281 10.1002/ijc.2910210305 631930

[B21] Banyard J Chung I Wilson AM Vetter G Le Béchec A Bielenberg DR Zetter BR Regulation of epithelial plasticity by miR-424 and miR-200 in a new prostate cancer metastasis model Sci Rep 2013 3 3151 24193225 10.1038/srep03151 PMC3818652

[B22] Chunthapong J Seftor EA Khalkhali-Ellis Z Seftor RE Amir S Lubaroff DM Heidger PM Jr Hendrix MJ Dual roles of E-cadherin in prostate cancer invasion J Cell Biochem 2004 91 4 649 661 10.1002/jcb.20032 14991757

[B23] Yilmaz M Christofori G Mechanisms of motility in metastasizing cells Mol Cancer Res 2010 8 5 629 642 10.1158/1541-7786.MCR-10-0139 20460404

[B24] Wong SY Hynes RO Lymphatic or hematogenous dissemination: how does a metastatic tumor cell decide? Cell Cycle 2006 5 8 812 817 10.4161/cc.5.8.2646 16627996 PMC1459485

[B25] Christiansen A Detmar M Lymphangiogenesis and cancer Genes Cancer 2011 2 12 1146 1158 10.1177/1947601911423028 22866206 PMC3411123

[B26] Datta K Muders M Zhang H Tindall DJ Mechanism of lymph node metastasis in prostate cancer Future Oncol 2010 6 5 823 836 10.2217/fon.10.33 20465393 PMC2892838

[B27] Yee DS Shariat SF Lowrance WT Maschino AC Savage CJ Cronin AM Scardino PT Eastham JA Prognostic significance of lymphovascular invasion in radical prostatectomy specimens BJU Int 2010 108 4 502 507 21050364 10.1111/j.1464-410X.2010.09848.x PMC4319653

[B28] Weidner N Carroll PR Flax J Blumenfeld W Folkman J Tumor angiogenesis correlates with metastasis in invasive prostate carcinoma Am J Pathol 1993 143 2 401 409 7688183 PMC1887042

[B29] Mumprecht V Detmar M Lymphangiogenesis and cancer metastasis J Cell Mol Med 2009 13 8A 1405 1416 10.1111/j.1582-4934.2009.00834.x 19583813 PMC3572232

[B30] Diamond E Lee GY Akhtar NH Kirby BJ Giannakakou P Tagawa ST Nanus DM Isolation and characterization of circulating tumor cells in prostate cancer Front Oncol 2012 2 131 23087897 10.3389/fonc.2012.00131 PMC3468833

[B31] Ni J Cozzi PJ Duan W Shigdar S Graham PH John KH Li Y Role of the EpCAM (CD326) in prostate cancer metastasis and progression Cancer Metastasis Rev 2012 31 3–4 779 791 22718399 10.1007/s10555-012-9389-1

[B32] Went P Vasei M Bubendorf L Terracciano L Tornillo L Riede U Kononen J Simon R Sauter G Baeuerle PA Frequent high-level expression of the immunotherapeutic target Ep-CAM in colon, stomach, prostate and lung cancers Br J Cancer 2006 94 1 128 135 10.1038/sj.bjc.6602924 16404366 PMC2361083

[B33] Poczatek RB Myers RB Manne U Oelschlager DK Weiss HL Bostwick DG Grizzle WE Ep-Cam levels in prostatic adenocarcinoma and prostatic intraepithelial neoplasia J Urol 1999 162 4 1462 1466 10.1016/S0022-5347(05)68341-3 10492238

[B34] Zellweger T Ninck C Bloch M Mirlacher M Koivisto PA Helin HJ Mihatsch MJ Gasser TC Bubendorf L Expression patterns of potential therapeutic targets in prostate cancer Int J Cancer 2005 113 4 619 628 10.1002/ijc.20615 15472903

[B35] Benko G Spajic B Kruslin B Tomas D Impact of the EpCAM expression on biochemical recurrence-free survival in clinically localized prostate cancer Urol Oncol 2013 31 4 468 474 10.1016/j.urolonc.2011.03.007 21514185

[B36] Denzel S Maetzel D Mack B Eggert C Barr G Gires O Initial activation of EpCAM cleavage via cell-to-cell contact BMC Cancer 2009 9 402 10.1186/1471-2407-9-402 19925656 PMC2784796

[B37] Thuma F Zoller M EpCAM-associated claudin-7 supports lymphatic spread and drug resistance in rat pancreatic cancer Int J Cancer 2013 133 4 855 866 10.1002/ijc.28085 23390083

[B38] Nubel T Preobraschenski J Tuncay H Weiss T Kuhn S Ladwein M Langbein L Zoller M Claudin-7 regulates EpCAM-mediated functions in tumor progression Mol Cancer Res 2009 7 3 285 299 10.1158/1541-7786.MCR-08-0200 19276185

[B39] Mercurio AM Rabinovitz I Shaw LM The alpha 6 beta 4 integrin and epithelial cell migration Curr Opin Cell Biol 2001 13 5 541 545 10.1016/S0955-0674(00)00249-0 11544021

[B40] Giancotti FG Targeting integrin beta4 for cancer and anti-angiogenic therapy Trends Pharmacol Sci 2007 28 10 506 511 10.1016/j.tips.2007.08.004 17822782

[B41] Yoshioka T Otero J Chen Y Kim YM Koutcher JA Satagopan J Reuter V Carver B de Stanchina E Enomoto K Greenberg NM Scardino PT Scher HI Sawyers CL Giancotti FG Beta4 Integrin signaling induces expansion of prostate tumor progenitors J Clin Invest 2013 123 2 682 699 23348745 10.1172/JCI60720 PMC3561800

[B42] Gerson KD Maddula VS Seligmann BE Shearstone JR Khan A Mercurio AM Effects of beta4 integrin expression on microRNA patterns in breast cancer Biol Open 2012 1 7 658 666 10.1242/bio.20121628 23213459 PMC3507297

[B43] Andreasen PA Kjoller L Christensen L Duffy MJ The urokinase-type plasminogen activator system in cancer metastasis: a review Int J Cancer 1997 72 1 1 22 10.1002/(SICI)1097-0215(19970703)72:1<1::AID-IJC1>3.0.CO;2-Z 9212216

[B44] Hienert G Kirchheimer JC Pfluger H Binder BR Urokinase-type plasminogen activator as a marker for the formation of distant metastases in prostatic carcinomas J Urol 1988 140 6 1466 1469 3193516 10.1016/s0022-5347(17)42074-x

[B45] Shariat SF Roehrborn CG McConnell JD Park S Alam N Wheeler TM Slawin KM Association of the circulating levels of the urokinase system of plasminogen activation with the presence of prostate cancer and invasion, progression, and metastasis J Clin Oncol 2007 25 4 349 355 10.1200/JCO.2006.05.6853 17264329

[B46] Miyake H Hara I Yamanaka K Gohji K Arakawa S Kamidono S Elevation of serum levels of urokinase-type plasminogen activator and its receptor is associated with disease progression and prognosis in patients with prostate cancer Prostate 1999 39 2 123 129 10.1002/(SICI)1097-0045(19990501)39:2<123::AID-PROS7>3.0.CO;2-2 10221568

[B47] Pulukuri SM Gondi CS Lakka SS Jutla A Estes N Gujrati M Rao JS RNA interference-directed knockdown of urokinase plasminogen activator and urokinase plasminogen activator receptor inhibits prostate cancer cell invasion, survival, and tumorigenicity in vivo J Biol Chem 2005 280 43 36529 36540 10.1074/jbc.M503111200 16127174 PMC1351057

[B48] Conn EM Botkjaer KA Kupriyanova TA Andreasen PA Deryugina EI Quigley JP Comparative analysis of metastasis variants derived from human prostate carcinoma cells: roles in intravasation of VEGF-mediated angiogenesis and uPA-mediated invasion Am J Pathol 2009 175 4 1638 1652 10.2353/ajpath.2009.090384 19729488 PMC2751560

[B49] Mamoune A Kassis J Kharait S Kloeker S Manos E Jones DA Wells A DU145 human prostate carcinoma invasiveness is modulated by urokinase receptor (uPAR) downstream of epidermal growth factor receptor (EGFR) signaling Exp Cell Res 2004 299 1 91 100 10.1016/j.yexcr.2004.05.008 15302576

[B50] O’Halloran TV Ahn R Hankins P Swindell E Mazar AP The many spaces of uPAR: delivery of theranostic agents and nanobins to multiple tumor compartments through a single target Theranostics 2013 3 7 496 506 10.7150/thno.4953 23843897 PMC3706693

[B51] Sanchez-Tillo E de Barrios O Siles L Amendola PG Darling DS Cuatrecasas M Castells A Postigo A ZEB1 Promotes invasiveness of colorectal carcinoma cells through the opposing regulation of uPA and PAI-1 Clin Cancer Res 2013 19 5 1071 1082 10.1158/1078-0432.CCR-12-2675 23340304

[B52] Drake JM Barnes JM Madsen JM Domann FE Stipp CS Henry MD ZEB1 coordinately regulates laminin-332 and {beta}4 integrin expression altering the invasive phenotype of prostate cancer cells J Biol Chem 2010 285 44 33940 33948 10.1074/jbc.M110.136044 20729552 PMC2962494

[B53] Gemmill RM Roche J Potiron VA Nasarre P Mitas M Coldren CD Helfrich BA Garrett-Mayer E Bunn PA Drabkin HA ZEB1-responsive genes in non-small cell lung cancer Cancer Lett 2010 300 1 66 78 20980099 10.1016/j.canlet.2010.09.007 PMC3337721

[B54] Park SM Gaur AB Lengyel E Peter ME The miR-200 family determines the epithelial phenotype of cancer cells by targeting the E-cadherin repressors ZEB1 and ZEB2 Genes Dev 2008 22 7 894 907 10.1101/gad.1640608 18381893 PMC2279201

[B55] Korpal M Lee ES Hu G Kang Y The miR-200 family inhibits epithelial-mesenchymal transition and cancer cell migration by direct targeting of E-cadherin transcriptional repressors ZEB1 and ZEB2 J Biol Chem 2008 283 22 14910 14914 10.1074/jbc.C800074200 18411277 PMC3258899

[B56] Gregory PA Bert AG Paterson EL Barry SC Tsykin A Farshid G Vadas MA Khew-Goodall Y Goodall GJ The miR-200 family and miR-205 regulate epithelial to mesenchymal transition by targeting ZEB1 and SIP1 Nat Cell Biol 2008 10 5 593 601 10.1038/ncb1722 18376396

