# Supplementary material for: Identification of genes regulating migration and invasion using a new model of metastatic prostate cancer
Source: BMC Cancer. 2014 May 30;14:387. doi: 10.1186/1471-2407-14-387 (PMC4046438; doi:10.1186/1471-2407-14-387)
Supplement: Additional file 1: Figure S1 — Heat map using Ingenuity Analysis software and the “Cell Signaling” category. The range of difference between DU145 and DU145LN4 groups are 1.8-16.4 fold. Table S1. Relative expression levels of PLAU, EPCAM, ITGB4 and housekeeping genes in the metastatic DU145-LN sublines. [file 1471-2407-14-387-S1.pptx]

## Slide 1
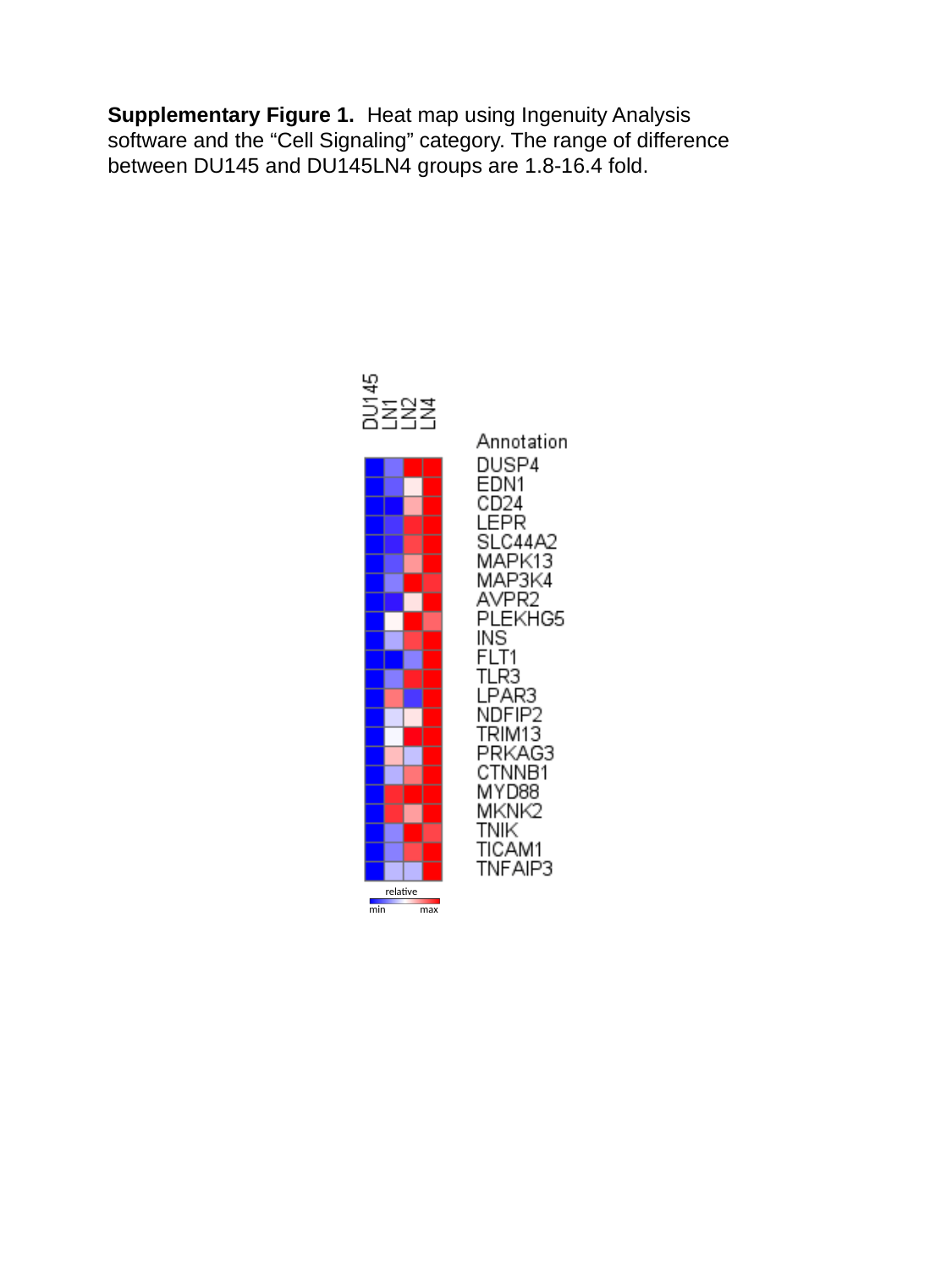

Supplementary Figure 1. Heat map using Ingenuity Analysis
software and the “Cell Signaling” category. The range of difference
between DU145 and DU145LN4 groups are 1.8-16.4 fold.
relative
min
max

## Slide 2
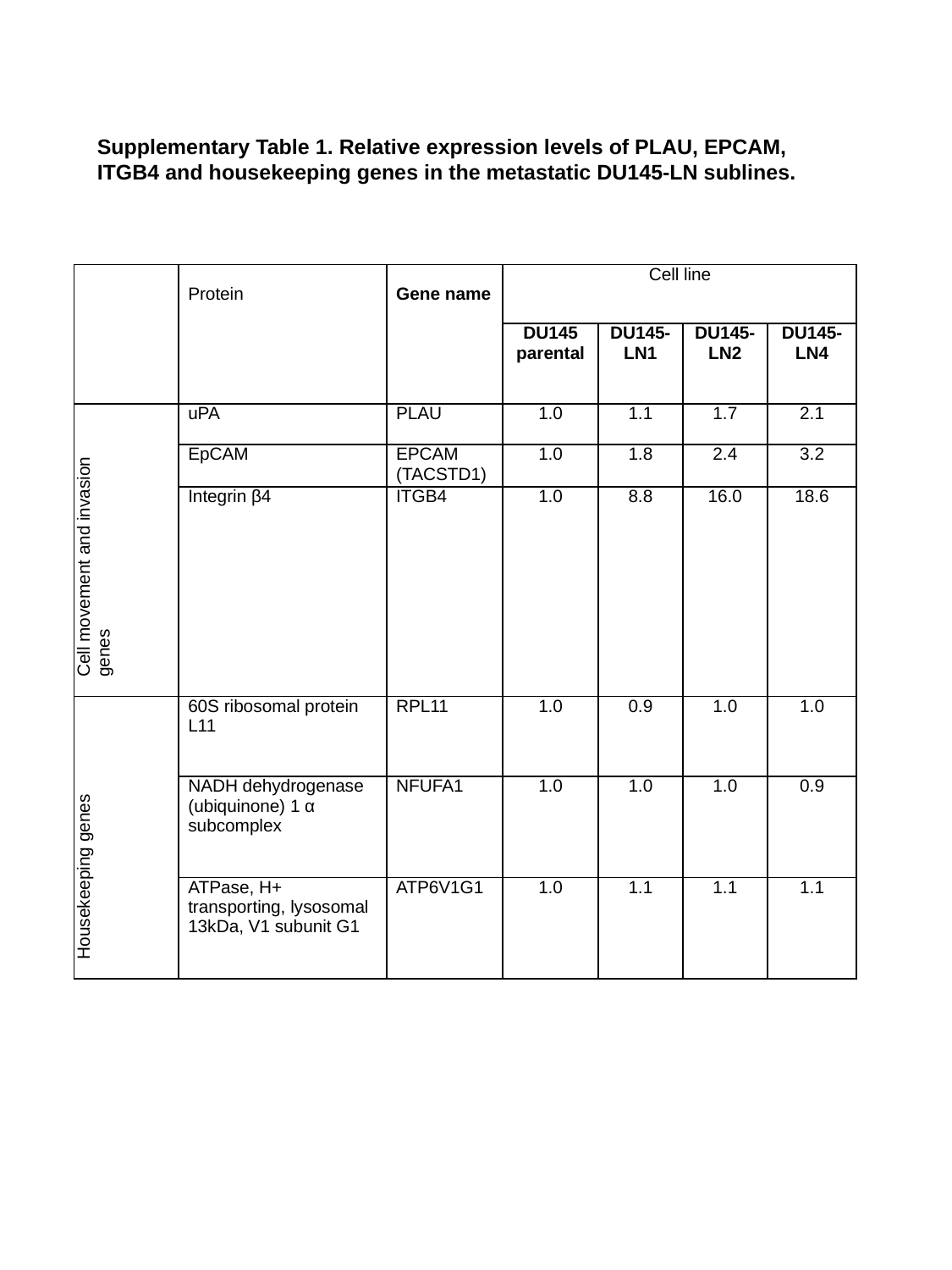

Supplementary Table 1. Relative expression levels of PLAU, EPCAM, ITGB4 and housekeeping genes in the metastatic DU145-LN sublines.
| | Protein | Gene name | Cell line | | | |
| --- | --- | --- | --- | --- | --- | --- |
| | | | DU145 parental | DU145- LN1 | DU145- LN2 | DU145- LN4 |
| Cell movement and invasion genes | uPA | PLAU | 1.0 | 1.1 | 1.7 | 2.1 |
| | EpCAM | EPCAM (TACSTD1) | 1.0 | 1.8 | 2.4 | 3.2 |
| | Integrin β4 | ITGB4 | 1.0 | 8.8 | 16.0 | 18.6 |
| Housekeeping genes | 60S ribosomal protein L11 | RPL11 | 1.0 | 0.9 | 1.0 | 1.0 |
| | NADH dehydrogenase (ubiquinone) 1 α subcomplex | NFUFA1 | 1.0 | 1.0 | 1.0 | 0.9 |
| | ATPase, H+ transporting, lysosomal 13kDa, V1 subunit G1 | ATP6V1G1 | 1.0 | 1.1 | 1.1 | 1.1 |
